# First episode of psychosis in patients over 35 years of age, the forgotten population. A descriptive and comparative study

**DOI:** 10.1192/j.eurpsy.2025.417

**Published:** 2025-08-26

**Authors:** G. Pérez Guerrero, R. Pérez Iglesias, J. Vázquez Bourgon, P. Suárez Pinilla, D. Sierra Biddle, M. L. Ramírez Bonilla, P. Cordero Andrés, M. D. C. Flor Gómez

**Affiliations:** 1 Psychiatry, Hospital Regional Universitario de Málaga, Málaga; 2 Psychiatry, Hospital Universitario Marqués de Valdecilla, Santander, Spain

## Abstract

**Introduction:**

Early Intervention in Psychosis (EIP) services have been youth-focused since their inception. Recently, NICE recommend the expansion of the age acceptability criterion to 65 years, from the previous cut-off of 35 years.

**Objectives:**

The aim of this study is to compare the demographic and clinical characteristics between patients below and above 35 years of an epidemiological cohort of first-episode psychosis patients treated in the Early Intervention Service of Cantabria (ITPCan).

**Methods:**

This is an study of the 207 consecutive patients aged from 17 to 65 who were admitted to the Service of University Hospital Marqués de Valdecilla, Spain, from January 2020 to July 2024. Descriptive statistics and between groups comparisons are reported.

**Results:**

A large proportion (51.2%) of those who presented a first episode of psychosis did so after the age of 35. The over 35s were predominantly female (65,1%), whilst the under 35s were predominantly male (58.4%) (χ2=11.495, P=0.01).

No significant differences were found between the age groups in terms of the need for psychiatric care in the different facilities (emergency care, day hospital, acute unit admission or mid-stay unit admission), nor in the presentation of autolytic attempts or the requirement for mechanical restraint.

DUP was significantly higher in the over 35s (u=4183.5, P=0.006), with a median of 4 months versus a median of 2 in the under 35s.

As for the use of drugs, 36.6% of the under 35s regularly consume cannabis compared to 6.6% of the over 35s (χ2=15.783, P=0), while there are no differences in the consumption of tobacco or alcohol.

There was a higher proportion of patients over 35 with diabetes (4.7% vs. 0%, χ2=4.407, P=0.036), hypercholesterolemia (20.8% vs. 4%; χ2=11.750, P=0.001) and a history of cardiovascular disease (7.5% vs. 1%, χ2=4.678, P=0.03), but no significant differences were observed between groups in the history of cerebrovascular disease or hypertension.

In reference to diagnosis, non-affective psychosis was more frequent in both groups (73.3% in under 35s and 90.6% in over 35s), with the diagnosis of delusional disorder being more frequent in the group over 35s (18.9% vs 1%, χ2=16.127, P=0). On the other hand, the diagnosis of manic episode with psychotic symptoms was significantly higher in those under 35 years of age (22.8% vs 9.4%, χ2=6.867, P=0.009).

**Conclusions:**

Despite the high proportion of patients who have a late onset of psychosis, specially women, this remains an understudied group. EIP services focused on young people are gender and age inequitable. There are some clinical and demographic differences between the two age groups and EIP services should ensure that the treatments offered are tailored to the needs of both groups.

**Disclosure of Interest:**

None Declared

